# Everybody's Day

**Published:** 1920-10-09

**Authors:** 


### The Hospital

The Workers' Newspaper of Administrative Medicine and Institutional Life, Administration, National Insurance and Health.

No. 1792, Vol. LXIX.; SATURDAY, OCTOBER 9, 1920. PRICE TWOPENCE

### EVERYBODY'S DAY.

Red Cross Week, which commences on October 9 and lasts until October 17, with its central feature, "Our Day," on Thursday, October 14, is at once a test and an opportunity. It is a test of the extent to which voluntary co-operation on the part of each member of the public in the work of caring for the sick is still a live thing. As to this we have never lost our faith, and it is our opinion that the present seeming apathy is the outcome of the enormous changes in the economic and social life of the people, and that personal desire to help and keen sympathy still lie close to the surface.

We cannot believe that the nation as a whole is unaware of the enormous extent of the field of work for the sick and disabled, largely a consequence of the war, which is crying aloud for helpers. Nor, after a flood of arguments, are we prepared to embrace a theory that the best method of dealing with this widespread condition is by means of machine-made ministrations of a municipality or Government department. Wise deliberations may, it is true, find that certain work can properly be undertaken by Government, Imperial or local, or at least be subsidised by public funds, but there are large fields which necessarily must remain untouched or uncovered by the work of public officials or by public finance. It is precisely in these fields that the huge organisation and invaluable prestige of the British Red Cross Society and Order of St. John of Jerusalem are likely to do most lasting and effective service, and the wisely chosen programme of the Joint Societies is exactly representative of the objects which are best dealt with by private enterprise and beneficence.

First, there is the care of sick and wounded ex-Service men, which very properly should be the joint affair of the public as represented by the Government and the public as represented by themselves, and not the single affair of either. To those whose privilege it has been to attend upon or visit the men who are still in our hospitals for injuries directly caused by the war, and have personally seen their extraordinary fortitude and cheerfulness it is unnecessary to appeal. They know as well as anyone can possibly know that what can be done for the happiness and welfare of these men will never solely be accounted for by a capitation grant.

The next object of the campaign, Maternity and Child Welfare, is one which it goes without saying needs all the peculiar sympathy of voluntary help and personal voluntary work far beyond anything which can be supplied through the excellent though insufficient organisation of local government. An argument that the upkeep of motor ambulances should also be an item in the programme of voluntary effort is largely dependent upon the fact that the success of this service under Red Cross management is so marked that it seems almost dangerous to take the work from such able hands. Moreover, this service is firmly linked with hospital, maternity, child welfare, and district nursing organisations, and could only with difficulty, even if it were consistent with the claims of efficiency, be disassociated from those fields.

A further principal object of Red Cross Week concerns the civil hospitals in London and the provinces, and in this movement the Joint Committee, as has been announced, will work hand-in-hand with such powerful organisations as the London Hospital Saturday Fund and the League of Mercy, distributing an appropriate part of the proceeds not, as was first intended, amongst provincial hospitals only, but amongst the hospitals of London as well. Up and down the country, at meetings of representatives of joint societies, and at gatherings to form the regional committees of the British Hospitals Association, Sir Arthur Stanley and Sir Napier Burnett have worked untiringly to demonstrate the needs of the sick in hospitals and the part that almsgiving and personal voluntary service must play in their alleviation.

Here, too, if it were merely a matter of cold finance, it has been brought home quite definitely that the voluntary system is the better business, a point of view recommended to the ratepayer in general and to employers, insurance companies, and others in particular. Roughly, we are in this position. The expenditure of hospitals has doubled since before the war; the income, taking in such additions as patients' payments, has increased, or is likely to increase, by about fifty per cent. In other words, about one quarter of the expenses of hospitals is uncovered by prospective income. That a part of the deficit will have to be made good by grants from public funds nobody can doubt. That these grants will come under the system suggested in the Ministry of Health Bill before Parliament, if a general consensus of opinion goes for anything, is unlikely. But the State is up to its neck in obligations already, and is not likely to foot the bill in its entirety, even if it were desirable for it to do so. Here, most appropriately, the Red Cross movement will help, and it is clear that on the success of "Our Day" the efficiency of our hospital service, at any rate for the present, depends.

Such are the objects of Red Cross Week, and of its central feature is "Our Day," or "Everybody's Day," as we have ventured to call it. We trust that the goal aimed at, the raising of one million pounds a year, will be attained.

But a wise view of the movement, and of the whole situation, cannot be altogether objective. We have said that a test and an opportunity are presented. The test we have alluded to; the opportunity is not exclusively in the act of giving; it is the subjective effect of that giving which to a spiritually sick nation is so important. Maeterlinck says that nothing matters except as to its ennobling or debasing effect on the soul. Although a consciousness of gain would almost mean loss, the subjective effect of a good action must ennoble the individual, and through him the community. Materially or spiritually, the opportunity, whichever way one looks at it, is invaluable. The citizen who neglects it runs great risks and shoulders grave responsibility. He will have missed something, and conceivably have marred much.

In the evening of life may we have more precious memories! To every one of us—those who can give with service of body and mind, those whose worldly position enables them to give also of their substance, strictly, fairly, and in accordance with their means, and not nominally or mechanically—Everybody's Day is a great and precious occasion. It is one, too, which, while affording material assistance to the cause of the sick, may, properly and fully used, serve to lift the cloud of apathy and selfishness which lowers over our land.

